# Arginine is a component of the ammonium-CYG56 signalling cascade that represses genes of the nitrogen assimilation pathway in *Chlamydomonas reinhardtii*

**DOI:** 10.1371/journal.pone.0196167

**Published:** 2018-04-23

**Authors:** David González-Ballester, Emanuel Sanz-Luque, Aurora Galván, Emilio Fernández, Amaury de Montaigu

**Affiliations:** Departamento de Bioquímica y Biología Molecular, Campus de Rabanales, Universidad de Córdoba, Córdoba, Spain; Universidad de la Laguna, SPAIN

## Abstract

Nitrogen assimilation and metabolism are essential processes for all living organisms, yet there is still much to be learnt on how they are regulated. The use of *Chlamydomonas reinhardtii* as a model system has been instrumental not only in identifying conserved regulation mechanisms that control the nitrogen assimilation pathway, but also in understanding how the intracellular nitrogen status regulates metabolic processes of industrial interest such as the synthesis of biolipids. While the genetic regulators that control the nitrogen pathway are successfully being unravelled, other layers of regulation have received less attention. Amino acids, for example, regulate nitrogen assimilation in certain organisms, but their role in Chlamydomonas has not thoroughly been explored. Previous results had suggested that arginine might repress key genes of the nitrogen assimilation pathway by acting within the ammonium negative signalling cascade, upstream of the nitric oxide (NO) inducible guanylate cyclase *CYG56*. We tested this hypothesis with a combination of genetic and chemical approaches. Antagonising the effects of arginine with an arginine biosynthesis mutant or with two chemical analogues released gene expression from ammonium mediated repression. The *cyg56* and related *non1* mutants, which are partially insensitive to ammonium repression, were also partially insensitive to repression by arginine. Finally, we show that the addition of arginine to the medium leads to an increase in intracellular NO. Our data reveal that arginine acts as a negative signal for the assimilation of nitrogen within the ammonium-CYG56 negative signalling cascade, and provide a connection between amino acid metabolism and nitrogen assimilation in microalgae.

## Introduction

Nitrogen is an essential macroelement whose assimilation and metabolism must be tightly regulated in order to coordinate changes in the intracellular nitrogen supply with the assimilation of other elements such as carbon. It is for this reason that the regulation of nitrogen assimilation has been intensively studied since many years in a broad range of organisms, including in microalgae. Throughout the years, the key transporters and enzymes involved in nitrogen uptake and assimilation have been characterized and were shown to be highly conserved [1]. How they are regulated, however, can differ among algal species. *Chlamydomonas reinhardtii* (Chlamydomonas hereafter) has been an important model species for the discovery of novel regulators that control genes and proteins of the nitrogen assimilation pathway [2]. This organism is particularly well suited for the generation of insertional mutant libraries [3], which have facilitated the identification of several transcriptional regulators of nitrogen pathway genes [4]. More recently, Chlamydomonas has been used as a model to explore the connection between nitrogen starvation and the accumulation of triacylglycerols (TAG), a potential source of biofuels [5,6]. Limiting the nitrogen supply could be an attractive way of increasing TAG accumulation, but a deeper understanding of how nitrogen starvation induces TAG accumulation is necessary to overcome some of the limitations of this approach [7,8]. In summary, many aspects of how microalgae regulate nitrogen assimilation are still poorly understood, and further exploring how they coordinate the intracellular nitrogen status with their cellular metabolism holds the promise of better exploiting their biotechnological potential.

When the two most common sources of inorganic nitrogen, ammonium and nitrate, are present in the extracellular medium, Chlamydomonas preferentially assimilates ammonium. Once inside the cell, ammonium strongly represses nitrate assimilation by repressing the expression and inhibiting the activity of key enzymes and transporters of the pathway [1,2]. The expression of nitrogen assimilation genes actually responds to a balance of both negative (ammonium) and positive (nitrate) signals [9]. The gene *NIA1* is central to the nitrate assimilation pathway as it codes for the enzyme nitrate reductase that catalyses the reduction of nitrate [10]. The expression of *NIA1* is almost undetectable in the presence of high concentrations of ammonium, even when the activating signal nitrate is simultaneously present in the medium [9,11]. But not all genes involved in nitrogen assimilation respond to ammonium and nitrate in the same way. The ammonium transporter gene *AMT1*.*1* is typically repressed in conditions where either ammonium or nitrate are present, but contrary to *NIA1* its expression is still detectable in these repressive conditions [12]. It was shown that nitrate mediated repression of *AMT1*.*1* partially requires intracellular conversion of nitrate to ammonium [12]. The expression of *AMT1*.*1* and *NIA1* respond very precisely to variations in the concentration of ammonium or nitrate, such that *AMT1*.*1* and *NIA1* transcript levels can be used as sensitive indicators of the capacity of cells to correctly sense nitrogen [9,10]. Due to their different regulation mechanisms and sensitivities to the inductive and repressive signals, one gene may be more suitable as a marker than another depending on the nitrogen context and experimental design [9,13].

The use of Chlamydomonas as a model system has been instrumental in the discovery of genes that regulate nitrogen assimilation. The positive regulator of nitrate assimilation NIT2 was originally isolated in Chlamydomonas [14], and its function was later shown to be conserved in higher plants [15,16]. Progress has also been made in the identification of genes that mediate repression by ammonium of nitrogen assimilation genes. The genes *CYG56* and *NO NITRATE 1* (*NON1*) were isolated from a mutant screen whose aim was to discover novel genes involved in the ammonium-mediated repression of *NIA1* [4]. CYG56 was shown to be an NO inducible guanylate cyclase, which led to the discovery of NO being a repressor of nitrogen assimilation genes in Chlamydomonas [11]. NON1, on the other hand, does not contain any known functional domains, and seems to be acting in the same NO dependent pathway than CYG56 [13]. It had been suggested that the amino acid arginine could be acting upstream of NO and CYG56 within the pathway, but this idea was largely hypothetical and lacked experimental support [11]. The hypothesis was based on arginine being a source of NO in numerous organisms. Ammonium triggers an increase in intracellular NO levels in Chlamydomonas [11], and ammonium is necessary for the synthesis of all amino acids including arginine. It was therefore proposed that ammonium might indirectly promote the synthesis of NO by increasing intracellular levels of arginine [11].

To date, the best-known example of an amino acid acting as a regulator of nitrogen assimilation is glutamine, which is a potent repressor of the nitrate assimilation pathway in plants [17,18]. Amino acids can be synthesised through different metabolic routes, and one of the major routes for the synthesis of amino acids is the Glutamine Synthase (GS) / Glutamine Oxoglutarate Aminotransferase (GOGAT) cycle. As glutamine is an important signalling molecule for nitrogen sensing in plants, the effect of inhibiting GS with methionine sulfoximine (MSX), an analogue of glutamate that interferes with GS activity, had previously been tested in Chlamydomnas cells grown in media containing ammonium, nitrate, or deprived of nitrogen [12]. The effect of MSX had been assessed by quantifying the expression of *AMT1*.*1*, whose transcript is detectable in the three conditions, but which is strongly repressed by nitrate and ammonium [12]. Application of MSX partially de-repressed *AMT1*.*1* in both ammonium and nitrate containing media, but had only a weak effect in medium deprived of nitrogen where *AMT1*.*1* transcript levels are high. These experiments showed that glutamine or other amino acids derived from glutamine likely act as negative signals on the expression of *AMT1*.*1*, and more broadly that amino acids are implicated in sensing nitrogen in Chlamydomonas. Ammonium and nitrate were nevertheless still capable of partially repressing *AMT1*.*1* expression in the presence of MSX, implying that ammonium and nitrate can act through other routes independent of GS/GOGAT.

Arginine is an important nitrogen storage molecule, among other reasons because the composition of this amino acid is rich in nitrogen [19,20]. It is a key signalling molecule in animal systems as it acts as a major activator of the mTORC1 kinase pathway [21]. Interestingly, Chlamydomonas possesses a specific transport system for arginine but not for any other amino acid [22]. A major goal of the current work was to test whether the amino acid arginine was a repressive signal for nitrogen assimilation in Chlamydomonas. We more specifically aimed to test whether arginine was implicated in the ammonium-CYG56 repressive signalling cascade, anticipating that it might act upstream of CYG56 in the pathway. These hypotheses were tested with a combination of genetic and chemical approaches. We first used the *arg7* mutant, which is not capable of synthesizing arginine, as a tool to ask whether alterations of the arginine biosynthesis pathway affected nitrogen signalling in Chlamydomonas cells. We further confirmed that chemical analogues of arginine had similar effects than the *arg7* mutation. We finally used the *cyg56* and *non1* mutants to determine if arginine acted upstream of *CYG56* and *NON1* to repress genes of the nitrogen assimilation pathway.

## Materials and methods

### Strains and conditions

**Fig 1:**
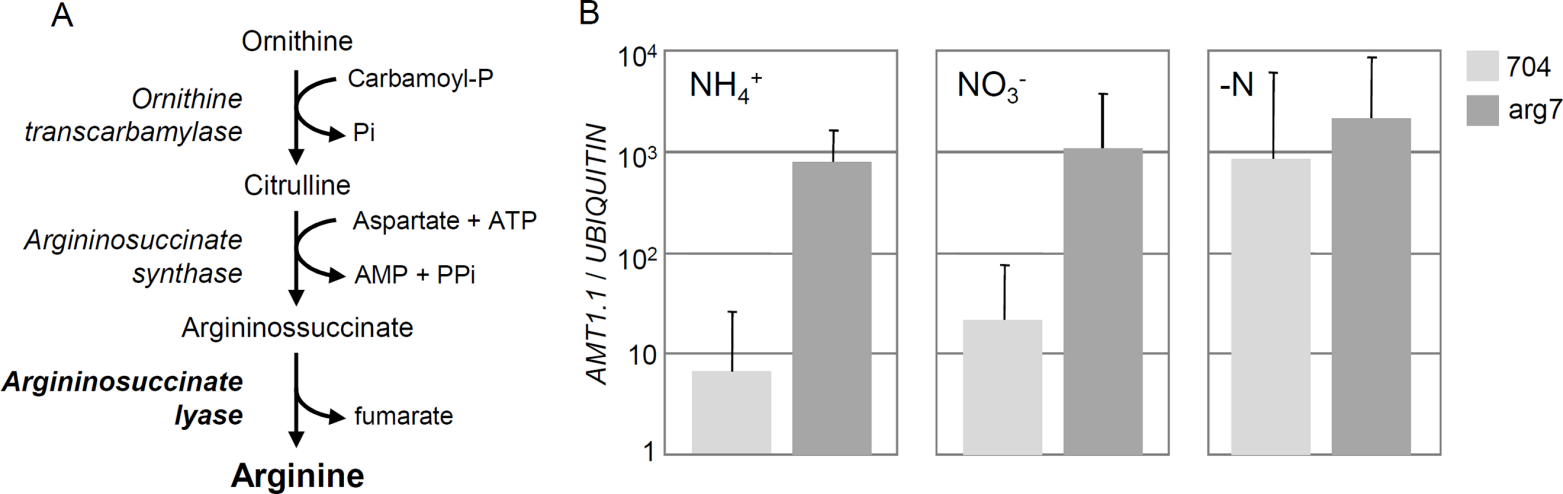
Disrupting the arginine biosynthesis pathway de-represses *AMT1*.*1* expression. (**A**). Representation of the three enzymatic reactions that lead to the synthesis of arginine from ornithine. Names of the enzymes are indicated in italics. (**B**). *AMT1*.*1* expression was measured in the *arg7* mutant and in the 704 control exposed to different nitrogen contexts (8 mM NH_4_^+^, 4 mM NO_3_^-^, or no nitrogen). Samples were collected after 1 hour in the different conditions. Error bars represent the standard deviation of at least three replicates from independent PCR runs. Cells were first left to grow for several days in medium containing 100 mg.L^-1^ of arginine and 8 mM NH_4_^+^ until they reached the exponential growth phase. Cells were then washed and transferred to nitrogen-free medium where they were exposed to different nitrogen sources as indicated on the figure.

With the exception of the arylsulfatase (ARS) tests, which were done on solid medium, all experiments were performed in liquid media. For experiments performed in liquid media, biomass was obtained before the start of the experiment by growing the cells in medium containing 8 mM NH_4_^+^. For the experiment including the arg7 mutant (Fig 1), which is auxotroph for arginine, cell biomass was obtained in medium containing 8 mM NH_4_^+^ and 100 mg.L^-1^ of L-arginine. When the cell cultures had reached exponential growth, cells were centrifuged and the nitrogen-rich medium removed. Cells were then washed two times in nitrogen-free medium before being transferred to the induction media containing the different sources of nitrogen and chemicals. Temperature and light settings of the growth chamber were 23°C and constant light (~75 μmol photons.m^-2^.s^-1^). Concerning the strains, *non1* and *cyg56* were obtained by insertional mutagenesis using 704 (cw15 arg7+ NIA1::ARS mt+) as the parental strain [4]. The plasmid used for the transformation contained the *APHVIII* gene conferring resistance to paromomycin. The *arg7* mutant contains a mutation in the argininosuccinate lyase (*ARG7)* gene that abolishes argininosuccinate lyase activity [23]. The 704 strain was originally obtained by complementation of *arg7* with a functional copy of the *ARG7* gene [24].

### Arylsulfatase (ARS) activity

All the strains used in this study bear the *ARS* gene as a reporter gene under the control of the *NIA1* promoter allowing us to use ARS activity as a proxy for *NIA1* promoter activity in different conditions. To test ARS activity, cells were grown for three or four days on solid agar medium containing 4 mM NO_3_^-^ and 15 and 25 mM of L-arginine. Because of the genetic background of these strains (cell wall mutants) most of the ARS enzyme is secreted into the agar media where the activity can be assayed. Cells were removed from the surface of the agar plate with a razor blade. 10 mL of the reaction mixture containing 100 mM Tris-HCl pH7.5, 10 mM Imidazole, 0.1 mg/ml Naphtyl sulfate and 1 mg/ml Ortho-dianisidine-tetrazotized (TOD) was applied directly to the solid medium as previously described [24], and the agar plates were placed on a shaker set at low rotation speed. Gentle and continuous shaking of the agar plates allowed for the reaction mix to be evenly spread on the surface of the medium. Even distribution of the reaction mix on the plate was necessary to make sure that results of the ARS test were homogenous across the plate surface. When the brown colour appeared after approximately 30 minutes, the reaction mixture was removed and digital photos of the plates were taken.

### Quantitative real-time PCR

*AMT1*.*1* relative mRNA accumulation was measured by quantitative real–time PCR after culture induction with different nitrogen sources and chemicals. After cell cultures reached exponential growth, cells were washed two times in nitrogen-fee medium and transferred to the induction media containing different chemicals and sources of nitrogen. Ammonium was added in the form of NH_4_Cl at a concentration of 8 mM, and nitrate was added in the form of KNO_3_^-^ at a concentration of 4 mM. L-NAME and canavanine were used at a concentration of 1 mM. After 1 hour induction, cells were centrifuged at 3000 x g, resuspended in a lysis buffer containing SDS, and thoroughly vortexed for several minutes. The composition of the lysis buffer was: 100 mM Tris-HCl (pH 8.0), 400 mM NaCl, 50 mM EDTA and 2% SDS. The phenol extraction method followed by precipitation with LiCl was used to isolate the RNA [25]. cDNA was synthesized with the Superscript II reverse transcriptase (Invitrogen) using an oligo(dT) primer and 1 μg RNA as template. The qRT-PCR reactions and fluorescence detection were performed with an iCycler iQ real-time PCR detection system (Bio-Rad). The fluorescent dye was SYBR® Green (Molecular Probes). Primers were designed with Primer Select (DNA Star Inc. v. 4.05). As in previous studies (9,11), and based on the ΔCt method described elsewhere [26,27], *AMT1*.*1* expression was normalized to *UBIQUITIN* expression by subtracting the CT value of *UBIQUITIN* from the CT value of *AMT1*.*1*. Primer sequences were as follows: AMT1.1fw GCACGGGAGGGCAAGAGGTTC, AMT1.1rev ATGTGCCGCAGTCAAGAAGGATTT, UBIfw GTACAGCGGCGGCTAGAGGCAC, UBIrev AGCGTCAGCGGCGGTTGCAGGTATCT.

### Fluorescence measurements with 4-Amino-5-methylamino-2',7'-difluorofluorescein diacetate (DAF-FM DA)

Chlamydomonas cells were grown in medium supplied with 4 mM NH_4_^+^ until they reached exponential growth. Following a previously described protocol [11], cells were then washed twice and incubated in medium without nitrogen before the application of the different sources of nitrogen. Arginine was added at the concentrations indicated on the figure, and nitrite was added in the form of KNO_2_ at a concentration of 10 mM. 1 μM DAF-FM DA probe (Invitrogen Molecular Probes) was applied 30 min before the measurements. Cells were washed, resuspended in 100 mM HEPES buffer pH 7.5, and transferred to 96-well back optiplates (Perkin-Elmer Life Sciences). The multidetection microplate reader (Synergy 4; Biotek) was used to detect fluorescence, with an excitation wavelength of 495 nm and an emission wavelength of 515 nm. Background fluorescence due to cell autofluorescence was determined by measuring fluorescence of a control sample without the DAF probe. Background fluorescence was then subtracted to total fluorescence measured in the samples exposed to different nitrogen sources. Three to eight technical replicates per condition were included on each plate.

## Results

The involvement of amino acids in the regulation of nitrogen assimilation was previously established by showing that MSX de-represses *AMT1*.*1* expression in the presence of ammonium and nitrate [12]. The first goal of the current manuscript was to build on these findings and test whether antagonising the effects of arginine with chemical analogues of arginine, or with a mutation that abolishes arginine biosynthesis, could alter *AMT1*.*1* expression in the same way than MSX. Arginine is produced by a metabolic pathway composed of eight enzymatic reactions that first lead to the synthesis of the ornithine intermediate, and then to the synthesis of arginine from ornithine (Fig 1A). The last step of the pathway is catalysed by the argininosuccinate lyase enzyme (ARG7) that converts argininosuccinate to arginine. The *arg7* mutant (also known as strain 325) bears a mutation that alters the activity of this enzyme [23], and we exploited this genetic resource to ask whether deficiencies in arginine biosynthesis could affect the expression of *AMT1*.*1*. In the arg7 mutant, *AMT1*.*1* expression levels were higher than in the 704 control strain (Fig 1B). However, the effect of *arg7* was much weaker in the absence of nitrogen, when no repressive signal for *AMT1*.*1* was present in the medium (Fig 1B). The repression mechanism of *AMT1*.*1* therefore requires the last step of the arginine biosynthesis pathway to be operational. These results support that arginine acts within the ammonium signalling cascade to repress *AMT1*.*1*. Arginine also seems to be implicated in nitrate-mediated repression of *AMT1*.*1*, which might at least partly be explained by intracellular conversion of nitrate to ammonium [12].

**Fig 2:**
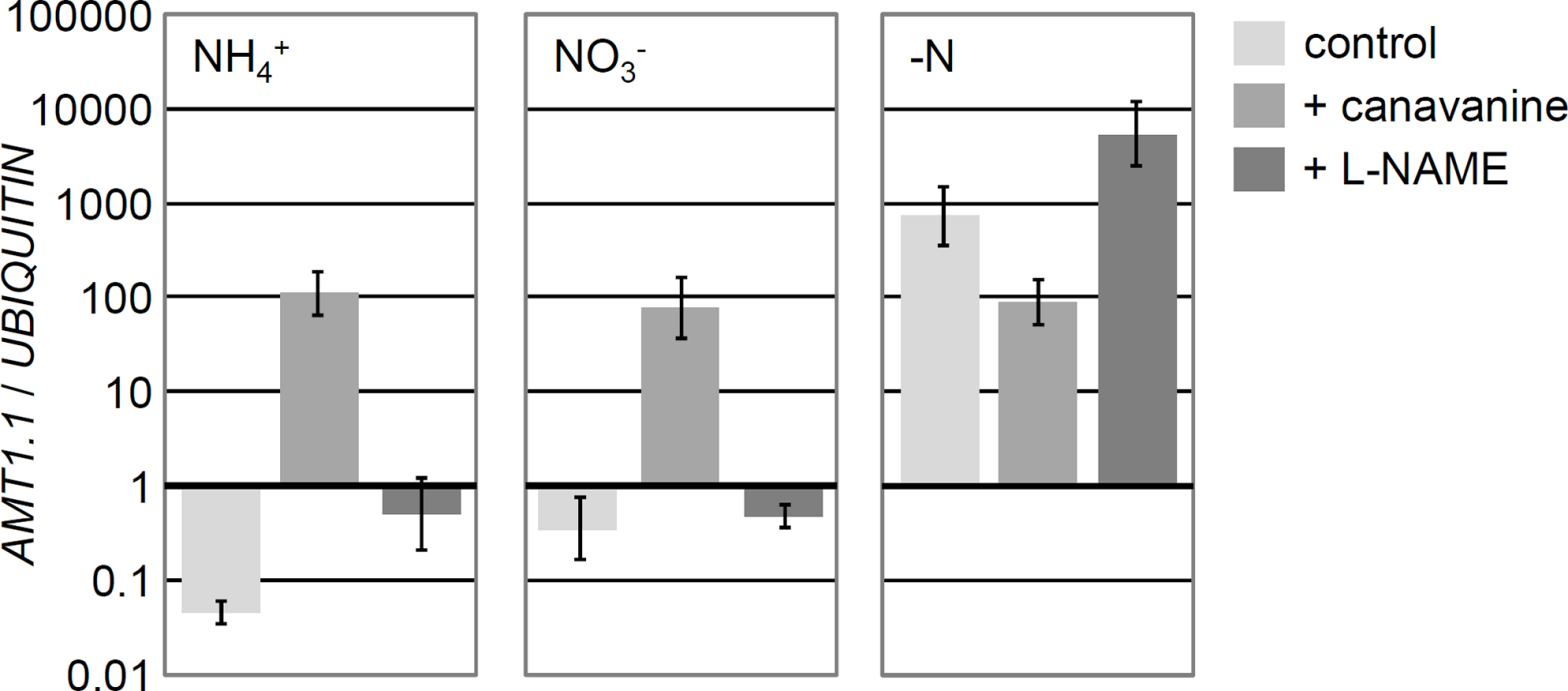
Chemicals that antagonise the effects of arginine de-repress *AMT1*.*1* expression in the presence of ammonium and nitrate. *AMT1*.*1* transcript levels were measured in wild type cells exposed to different nitrogen contexts (8 mM NH_4_^+^, 4 mM NO_3_^-^, or no nitrogen), and in the presence / absence of 1mM canavanine and 1mM L-NAME. Samples were collected after 1 hour in the different conditions. Error bars represent the standard deviation of at least three replicates from independent PCR runs. Cells were first left to grow for several days in medium containing 8 mM NH_4_^+^ until they reached the exponential growth phase. Cells were then washed and transferred to nitrogen-free medium where they were exposed to different nitrogen sources and chemicals as indicated on the figure.

We next used a chemical approach to further support that arginine was a repressor of *AMT1*.*1*. N(G)-Nitro-L-arginine methyl ester (L-NAME) and canavanine are chemical analogues of arginine that have been widely reported in different biological systems to inhibit processes in which arginine is involved [28,29]. Application of L-NAME and canavanine to the culture medium mimicked the effect of the *arg7* mutation on *AMT1*.*1* (Fig 2). The chemicals generally de-repressed *AMT1*.*1* expression in the presence of the repressive signals ammonium and nitrate (Fig 2). An exception was the mild effect of L-NAME in the presence of nitrate, but in the presence of ammonium this chemical promoted *AMT1*.*1* to levels 10 fold higher compared to the control (Fig 2). Canavanine was a stronger de-repressor than L-NAME in medium with ammonium or nitrate, yet it unexpectedly seemed to act as a repressor in the absence of nitrogen. The increase of *AMT1*.*1* expression in nitrogen-free medium supplied with L-NAME was more consistent with the results observed in ammonium and nitrate (Fig 2). Contrasting results of canavanine and L-NAME in the absence of nitrogen, together with the stronger effect of canavanine in medium with ammonium or nitrate suggest that canavanine might act through additional routes than L-NAME. Opposite outcomes of treatments with canavanine and L-NAME have been reported in the literature [30], and were explained by the different mode of actions of the chemicals. Our experiments describe for the first time the effects of L-NAME and canavanine in various nitrogen contexts, and the results of the L-NAME treatment in ammonium are consistent with the results of a previous experiment with L-NAME in media containing high ammonium concentrations [11]. Notwithstanding discrepancies in the nitrogen-free condition, the data overall support the analysis of the *arg7* mutant in that arginine deprivation mainly leads to de-repression of *AMT1*.*1* expression in medium with ammonium or nitrate. This strengthens the idea that arginine acts within the ammonium signalling cascade to repress *AMT1*.*1* expression.

**Fig 3:**
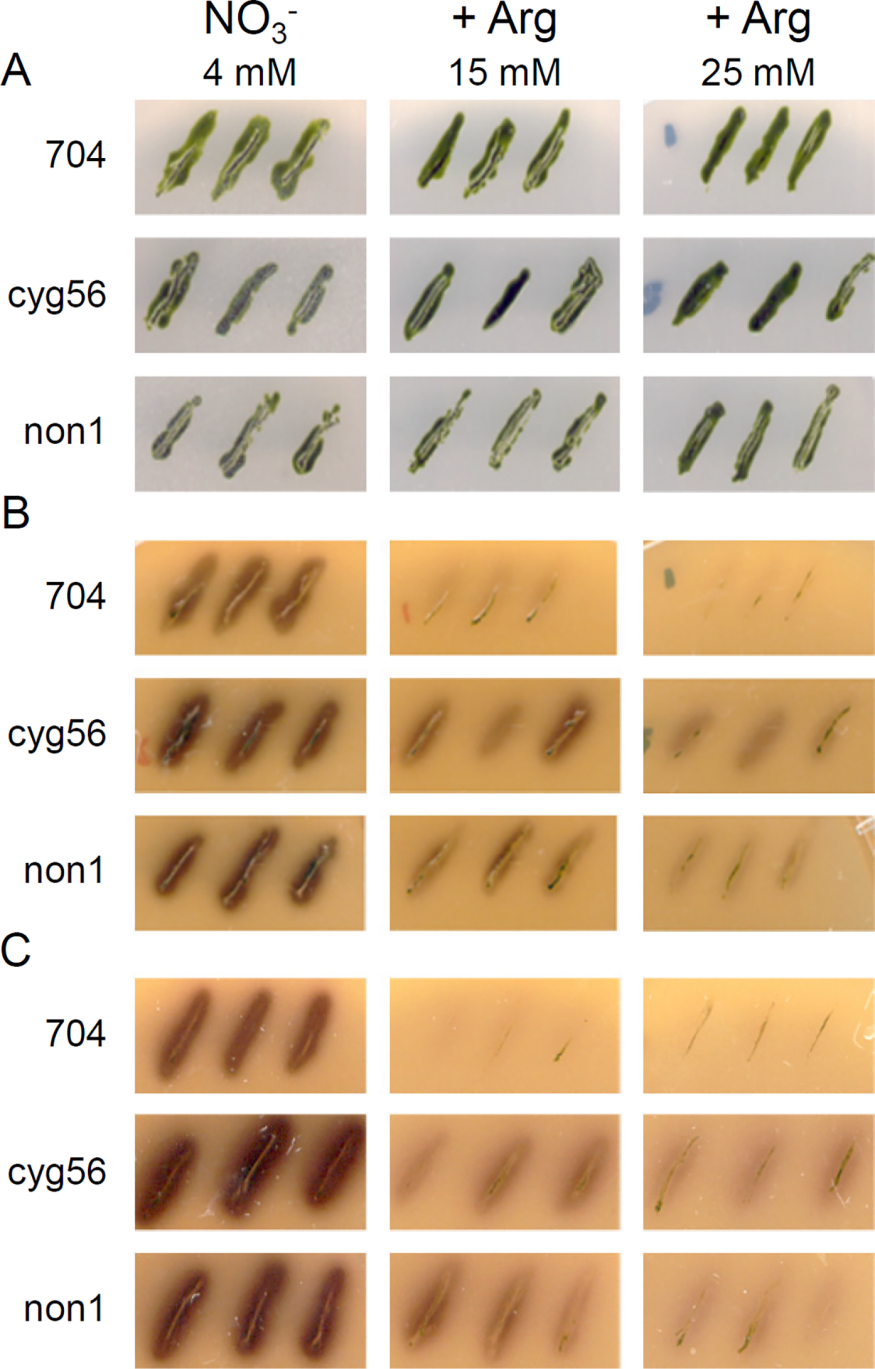
Arginine represses *NIA1* expression by acting within the *CYG56* pathway. Arylsulfatase (ARS) activity in the presence or absence of arginine was determined in the parental strain 704, and in the *cyg56* and *non1* mutants. NIA1::ARS activity was measured after three and four days of growth on solid medium supplied with 4 mM NO_3_^–^ and with different concentrations of arginine as indicated on the figure. (**A**). Growth control showing that the cells grew normally in the different conditions used in this experiment. (**B**). ARS test after three days of growth. (**C**). ARS test after four days of growth. The three strains 704, *cyg56* and *non1* bear a copy of the *ARS* gene fused to the *NIA1* promoter. ARS activity in the presence of arginine reveals that the *NIA1* promoter is not fully sensitive to arginine-mediated repression.

Ammonium represses genes of the nitrogen assimilation pathway at least partly by promoting the activity of CYG56 and NON1 [11,13]. *CYG56* codes for an NO-dependent guanylate cyclase, and *NON1* is a gene of unknown function that seems to be acting in the same pathway than *CYG56* [11,13]. If arginine acts in the same pathway than ammonium to repress nitrogen assimilation genes, it might do so by acting through *CYG56* and *NON1*. This idea was addressed with genetic experiments that aimed to test whether arginine signalling required *CYG56* and *NON1* activity [11,13]. The *cyg56* and *non1* mutants were isolated from a mutant screen designed to isolate strains deficient in their capacity to correctly repress *NIA1* promoter activity when ammonium was present in the medium [4]. *cyg56* and *non1* bear a construct composed of the arylsulfatase (*ARS*) reporter gene fused to the *NIA1* promoter that allows sensitive detection of *NIA1* promoter activity [4]. When the *NIA1* promoter is not fully repressed, the ARS enzyme is progressively excreted in the extracellular medium where its activity is detectable with a simple assay [24]. Before addressing whether arginine signalling required *CYG56* and *NON1* activity, we first tested if arginine could repress *NIA1*::*ARS*. The 704 control transformed with *NIA*::*ARS* showed almost no ARS activity when arginine was added to the medium, indicating that *NIA1* promoter activity was strongly inhibited by arginine (Fig 3B and 3C). Only a very faint NIA1::ARS signal was detectable when 15 mM of arginine was present, which shows that arginine was not saturating at this concentration (Fig 3B and 3C).

Finally, we tested if *CYG56* and *NON1* were acting in the same repressive pathway than arginine. 704 transformed with *NIA*::*ARS* is the parental strain of *cyg56* and *non1* [4], and was the adequate control for this experiment. Contrary to what was observed in the 704 control, NIA1::ARS was active in *cyg56* and *non1* grown in the presence of arginine (Fig 3A, 3B and 3C). This demonstrates that functional *CYG56* and *NON1* are required for arginine signalling to be operational, and therefore that arginine, *CYG56* and *NON1* act in the same pathway to repress *NIA1*.

**Fig 4:**
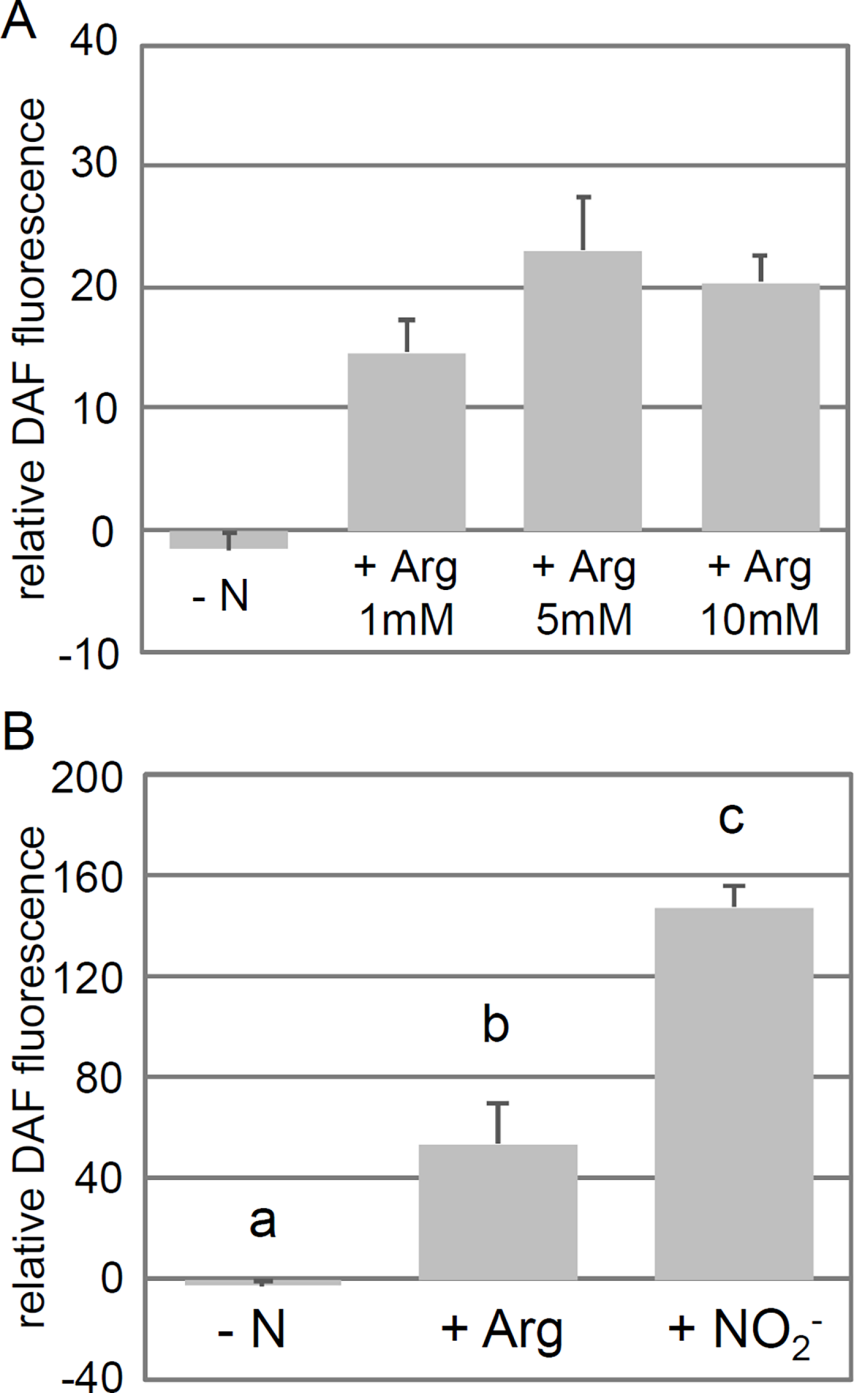
Application of arginine leads to an increase in DAF-FM DA fluorescence. Cells of the 704 strain were left to grow for several days in medium containing 8 mM NH_4_^+^ until they reached the exponential growth phase. Cells were then washed and transferred to nitrogen-free medium where they were exposed for one hour to different nitrogen sources as indicated on the figure. Background fluorescence due to autofluoresence was determined in a control without DAF-FM DA and was subtracted to the total fluorescence measured in the samples. (**A**). DAF-FM DA fluorescence measured after exposure to different concentrations of arginine. (**B**). DAF-FM DA fluorescence measured after exposure to 10 mM of Arginine and 10 mM of NO_2_^-^. Error bars represent the standard error from at least three independent measurements in (A), and at least seven independent measurements from two experiments in (B). Statistical differences between nitrogen treatments in (B) was determined with a one way ANOVA followed by multiple group comparison with the Tukey test. There was a significant effect of the nitrogen source on DAF-FM DA fluorescence (P<0.001, α = 0.05), and letters indicate the different statistical groups.

Arginine has been reported to be a source of NO in a variety of organisms. CYG56 is an NO-dependent guanylate cyclase, and *cyg56* and *non1* are both partially insensitive to NO [11,13] and to arginine (Fig 3). Based on these observations, it could be that arginine acts as a repressor by increasing intracellular levels of NO. However, evidence for arginine being a source of NO is still lacking in Chlamydomonas. We tested if the addition of arginine in the medium could lead to an increase in DAF-FM DA fluorescence, a probe used to quantify intracellular levels of NO in numerous biological systems including Chlamydomonas [11,31–34]. The addition of arginine in the extracellular medium effectively enhanced DAF-FM DA fluorescence in independent experiments (Fig 4A and 4B), supporting the idea that arginine acts upstream of CYG56 and NON1 by increasing NO levels. Another well-known source of NO in many organisms is nitrite, and a nitrite-dependent NO synthesis mechanisms has recently been described in Chlamydomonas [31]. We compared DAF-FM DA fluorescence levels resulting from the addition of nitrite and arginine, and found that nitrite produced a stronger signal (Fig 4B). So although arginine does trigger an increase in NO levels, our results raise the question of whether arginine-independent NO synthesis mechanisms are more efficient sources of NO. Nevertheless, the results of the ARS and DAF-FM DA experiments support that arginine at least partly acts upstream of CYG56 and NON1 in the ammonium signalling cascade, which was the goal of the current study.

## Discussion

The assimilation of nitrogen is a fundamental process for all living organisms, and its manipulation holds potential for industrial applications. The regulation mechanisms that control nitrogen assimilation are nevertheless far from being fully understood, notably in microalgae. The current work provides a contribution to the understanding of how amino acids, and more precisely arginine, regulate nitrogen assimilation in Chlamydomonas. The idea of arginine being a regulator of the nitrogen assimilation pathway arose based on a previous study in which it was speculated that arginine could be acting upstream of *CYG56* within the ammonium signalling cascade [11]. This hypothesis, however, lacked experimental support. Here, we assessed the role or arginine in the regulation of the nitrogen assimilation pathway with genetic and chemical approaches. Altogether, the application of chemical analogues of arginine and the analysis of the *cyg56*, *non1* and *arg7* mutants demonstrated that arginine is indeed a repressive signal for nitrogen assimilation genes. Consistent with previous assumptions [11], our results confirmed that arginine acts upstream of *NON1* and *CYG56*.

**Fig 5:**
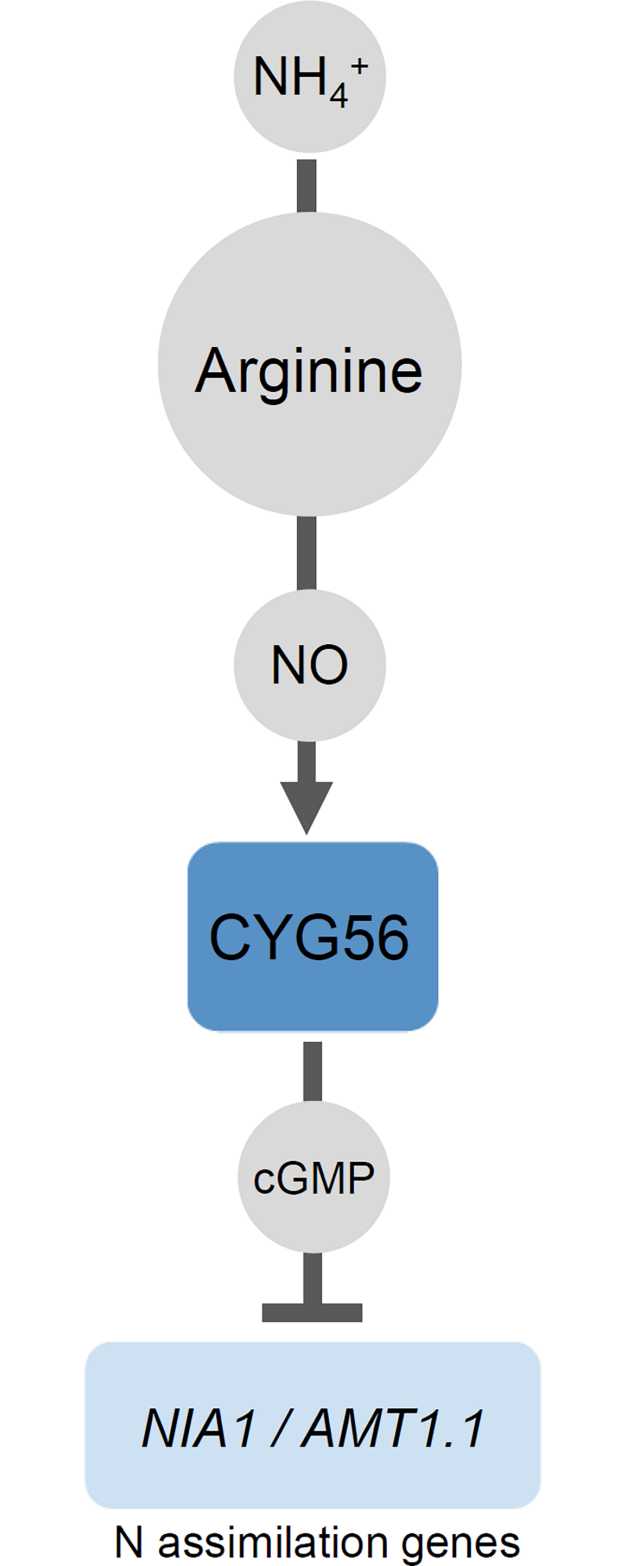
Scheme representing the involvement of arginine in the ammonium-*cyg56* signalling cascade. Grey circles represent signalling molecules. Dark blue boxes represent proteins, and light blue boxes represent genes. The scheme shows that a rise in intracellular ammonium leads to a rise in arginine and NO through mechanisms that remain to be characterized. NO promotes CYG56 activity by binding to its heme domain, resulting in an increase of cGMP levels that repress *NIA1* and *AMT1*.*1* [11].

The proposed implication of arginine in the ammonium-CYG56 signalling cascade is described in the scheme presented in Fig 5. The data from the *arg7* mutant (Fig 1) support that arginine acts downstream of ammonium in the pathway. NH_4_^+^ integration to carbon skeletons is an essential step of amino acid biosynthesis, but the metabolic route that synthesizes arginine within the pathway described here remains to be determined. Higher levels of arginine would then trigger an increase in intracellular NO through a yet unknown mechanism. The next step of the pathway is the binding of NO to the heme domain of CYG56 which, as previously demonstrated [11], stimulates its guanylate cycle activity. The resulting rise in cGMP levels finally represses genes of the nitrogen assimilation pathway. Importantly, it is not yet clear whether this is the only pathway through which arginine acts. The *cyg56* and *non1* mutations only partially relieved *NIA1*::*ARS* from arginine-mediated repression, suggesting that arginine might also act through other routes. Partial insensitivity of *cyg56* and *non1* had previously been observed in response to ammonium [11,13], and it had already been proposed that ammonium was acting by controlling various pathways [9]. Whether arginine and ammonium act together within pathways independent of CYG56 and NON1 remains to be determined. Our results nonetheless indicate that ammonium and arginine at least partially act together in a pathway involving CYG56 and NON1 to repress genes of the nitrogen assimilation pathway.

The experiments described in this work establish arginine as a component of the same repressive pathway than ammonium and *CYG56*, but they do not address how arginine acts to repress nitrogen assimilation genes. The data nonetheless provide some indications on how arginine-mediated repression might occur. CYG56 is a guanylate cyclase whose activity is promoted upon binding of NO to its heme domain. NO represses the expression of nitrogen assimilation genes, and CYG56 activity is required for NO to exert its repressive effect [11]. In many organisms, NO can be synthesised by the Nitric Oxide Synthase (NOS) enzyme that uses arginine as a substrate. According to our results, a possible scenario is that a hypothetical Chlamydomonas NOS uses arginine to synthesise NO, which then represses nitrogen assimilation genes by activating CYG56. There are many issues to be solved before this scenario is validated, the most important being that the presence of NOS has not been demonstrated in Chlamydomonas. Unlike *Ostreococcus tauri* and other algae [35], Chlamydomonas does not possess a homolog of animal NOS in its genome [36]. So although our results support that arginine is involved in the NO-mediated repression of nitrogen assimilation genes, it cannot be assumed that arginine-mediated repression of gene expression is due to NOS activity until an equivalent enzyme has been identified in Chlamydomonas. Even if an NOS-like enzyme does exist in Chlamydomonas, our results raise the question of whether nitrite is a more important source of NO. Efficient NO synthesis was recently shown under photosynthetic growth by a reductive process from nitrite, which involves the interaction in a dual system of the reductive half of nitrate reductase and the molybdoenxyme ARC (Amidoxime Reducing Component) [31]. In any case, the goal of this study was not to substantiate the existence of NOS in this alga, but to demonstrate that arginine was acting in the ammonium-CYG56 signalling cascade that represses genes involved in nitrogen assimilation.

Interestingly, this report is not the first to establish a connection between nitrogen sensing and arginine biosynthesis. PII signalling proteins are well-known sensors of the intracellular nitrogen status, and they control arginine biosynthesis by modulating the activity of N-acetyl-l-glutamate kinase [37]. Our data therefore strengthen the connection between nitrogen sensing and arginine biosynthesis by showing that arginine regulates the expression of key genes of the nitrogen assimilation pathway. Because NO-mediated regulation of nitrogen assimilation exists in other organisms [11,38,39], it will be worthwhile to test whether the role of arginine is conserved across species. Considering the metabolic diversity among microalgae, this might not always be the case. Of particular relevance would be to test if arginine-mediated regulation of nitrogen assimilation is present in *Ostreococcus tauri*, and whether it requires NOS activity. From a broader perspective, exploring the roles of arginine in microlalgae might lead to novel ways of manipulating the accumulation of compounds of industrial potential. Zalutskaya and co-authors (2015) proposed that TAG accumulation might be promoted by arginine deprivation independently of PII [40]. Thus, in addition to providing deeper insights into how nitrogen assimilation is regulated, further studying arginine signalling might also provide a better understanding of how TAG accumulation is regulated in Chlamydomonas.
